# Passive Fingerprinting of Same-Model Electrical Devices by Current Consumption

**DOI:** 10.3390/s23010533

**Published:** 2023-01-03

**Authors:** Mikhail Ronkin, Dima Bykhovsky

**Affiliations:** 1 Engineering School of Information Technologies, Telecommunications and Control Systems, Ural Federal University, 620078 Yekaterinburg, Russia; 2 Electrical and Electronics Engineering Department, Shamoon College of Engineering, Beer-Sheva 8410802, Israel

**Keywords:** device fingerprinting, consumption analysis, electrical network, electrical device, time-series classification (TSC), current measurement, switched-mode power supply (SMPS)

## Abstract

One possible device authentication method is based on device fingerprints, such as software- or hardware-based unique characteristics. In this paper, we propose a fingerprinting technique based on passive externally measured information, i.e., current consumption from the electrical network. The key insight is that small hardware discrepancies naturally exist even between same-electrical-circuit devices, making it feasible to identify slight variations in the consumed current under steady-state conditions. An experimental database of current consumption signals of two similar groups containing 20 same-model computer displays was collected. The resulting signals were classified using various state-of-the-art time-series classification (TSC) methods. We successfully identified 40 similar (same-model) electrical devices with about 94% precision, while most errors were concentrated in confusion between a small number of devices. A simplified empirical wavelet transform (EWT) paired with a linear discriminant analysis (LDA) classifier was shown to be the recommended classification method.

## 1. Introduction

There currently exist a number of powerful techniques for physical device fingerprinting, i.e., techniques for remote indirect identification or classification of a device of interest with or without the active cooperation of the fingerprinted device. The particular challenge faced by these techniques is related to the fingerprinting of similar devices, e.g., different devices that utilize similar hardware (same electrical scheme) and/or software, or use the same standardized communication protocols.

The fingerprinting task has been a subject of interest for many years. For example, remote identification of operating systems by the analysis of clock skews of the same communication protocol was provided in [1]. Ji et al. [2] showed effective fingerprinting of laptops with the same software and hardware by utilizing slight differences in the CPU’s emission of magnetic induction. It was shown that differences in the power consumption profiles of a mobile phone might be used to identify the currently used application [3]. The performance may be further improved when power consumption profiling is performed together with network traffic data analysis  [4].

Another area of considerable interest is the analysis of the current consumption of a hardware device. In this case, by measuring those currents, certain side-channel [5] and covert-channel [6,7] attacks are possible.

In this paper, we have addressed the fingerprinting of electrical devices with the same software and hardware by exploiting the microscopic deviations in current consumed from the electrical network. Such a fingerprinting technique has a few interesting possible applications. One of them is identifying counterfeit devices, which are expected to have significantly different fingerprints from authentic devices. While different-model devices monitoring is already commercially implemented (e.g., in [8]), same-model monitoring is still a challenging problem. The effective same-device fingerprinting solution may be expanded to electrical-network-based intrusion detection systems, which can check the fingerprints of all electrical-network-connected devices. Such a system may identify the special case of a supply-chain attack when a legitimate device is replaced with a malicious non-legitimate one. Such a system may also identify new devices with unknown fingerprints. This is particularly important in high-security facilities, where such a device may be part of a cyber-attack or a spyware device.

The main challenges and the impact of the paper related to the fingerprinting of electrical devices by examining the consumed current are as follows.

- All devices have exactly the same hardware and software and, therefore, similar consumption profiles.
- We have chosen relatively simple devices (computer monitors) with a single mode of operation. This is in contrast to computers [2] or cyber-physical systems (CPS) [9,10], where differences between complex modes of operation may be identified.
- The proposed measurement is completely passive, with all the devices having a similar unsupervised mode of operation. This is in contrast to the situation with dedicated supervision, such as a computer system that runs on particular calculations [2,6,7].
- Devices of interest do not include multiple feature-rich protocols, such as in the field of fingerprinting communications devices [11,12,13].
- A very low-frequency sampling rate of 50 kHz was applied. This sampling frequency is below the commonly applied bandwidth in RF-based fingerprinting [14] and is four times smaller than the one reported in the magnetic induction fingerprinting study reported in [2]. It is also lower than the switching frequency of common switched-mode power supplies (SMPSs) (see also Section 2 below).

The goal of the paper is to show that, in general, it is possible to discriminate between multiple devices of the same model by their current consumption. The preliminary results were published in [15].

The sampled current measurements are used as inputs to a time-series classifier. The following experimental results show the feasibility of device fingerprinting based on the consumed current analysis. The novelty of this work lies in its evaluation of modern time-series classification-based fingerprinting techniques. To the best of our knowledge, the given task has never been attempted under the constraints described above. Among the considered methods, the algorithm based on the simplified empirical wavelet transform (EWT) paired with a linear discriminant analysis (LDA) classifier was shown to be the recommended solution for this task.

The rest of the paper is organized as follows. Section 2 describes SMPS essentials. Section 4 provides experimental details. Section 3 reviews recent progress in time-series classification (TSC). Section 5 evaluates TSC of the experimental results followed by discussion in Section 6. Section 7 concludes and provides recommendations for future work.

## 2. Switch-Mode Power Supply Background

An SMPS is a basic component in modern computer-related devices and device fingerprinting heavily depends on SMPS properties. In this section, we describe the principle of SMPSs and then explain how SMPSs may be differentiated following their design principles.

### 2.1. Basic Principles

Consumer electronics and computer-related devices require stable and efficient DC voltage and current supplies. The modern approach is to convert from an AC electrical network to DC by a supply that is based on switching-regulator principles. A high-frequency switching regulator uses a series switching element that charges and discharges capacitors and/or inductors at sufficiently high frequency.

**Figure 1:**
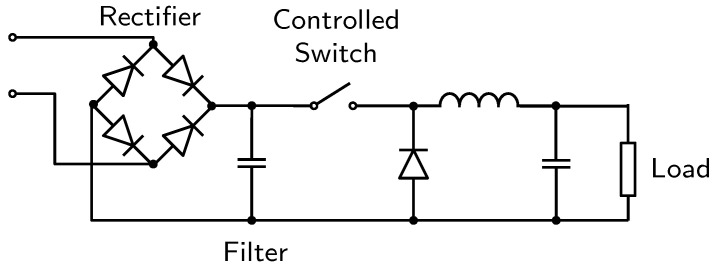
A simplified illustration of the switching regulator principle for AC–DC conversion.

An illustration of the generic SMPS design is presented in Figure 1. First, AC input is converted into DC by a rectification process using a rectifier and a filter. Then, the switch controls the charge and discharge of an LC circuit through the load. Moreover, the combination of the inductor and the capacitor form an LC filter that smooths out any ripple created by the switching action of the transistor. The typical switching frequency is between a few tens of kilohertz up to about one hundred kilohertz.

### 2.2. Electromagnetic Interference Filtering

**Figure 2:**
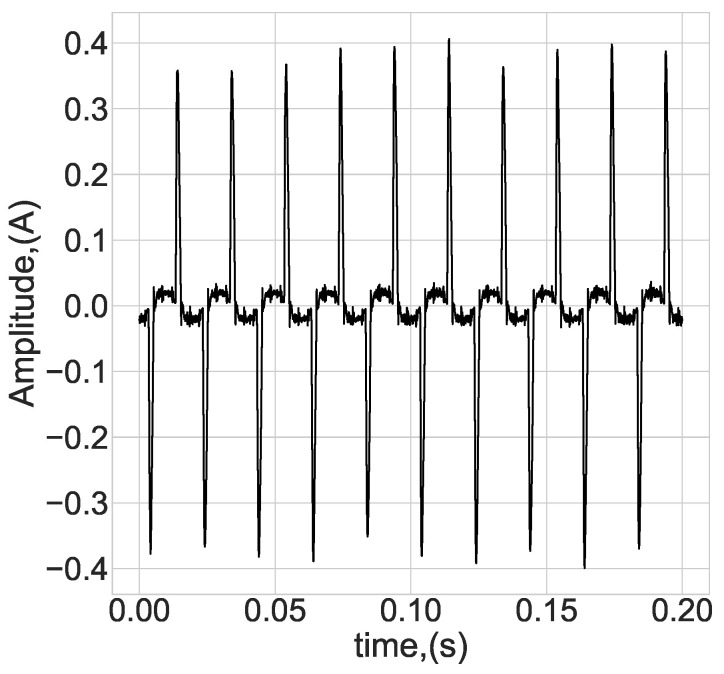
Illustration of the typical current consumption of SMPSs.

The current consumed from an electrical network is highly non-linear, as illustrated in Figure 2. Combining switching and non-linear consumption yields a wideband harmonic signal that may conduct to the electrical network [16]. The combination of a switching process and non-linear consumption produces a wideband harmonics signal that may conduct towards the electrical network. All power supplies contain electromagnetic interference (EMI) filtering to reduce conducted emissions toward the electrical network. This filtering is regulated by safety standards, such as FCC part 15 in the USA and CISPR 22 in Europe. According to common standards, the EMI filter dramatically reduces higher-frequency current components at frequencies higher than 150 kHz.

## 3. Time-Series Classification Considerations

Device fingerprinting involves the classification of signal segments, also termed TSC. In the following, we review current state-of-the-art TSC methods. The TSC methods can be divided into four groups, as discussed in the following sub-sections. In general, these methods reflect the performance-complexity trade-off that refers to the balance between the classification efficiency of a method, and the amount of resources (such as time, memory, or computational power) required to achieve that efficiency.

### 3.1. Data-Based Approach

The data-based methods are applied directly to raw or easily transformed time-series segments. In this approach, every two series, or their segments, are compared either as vectors (as with traditional multidimensional data points) or by some distance measure that uses all data points [17]. The segments can be selected pseudo-randomly, by some predefined rules or by their discriminative power (like shapelets). Most of these methods are applied in the time domain; some of them also incorporate the frequency or the pseudo-frequency domain [18,19]. Typically, distance measures are elastic, such as dynamic time-warping (DTW) distances.

The main disadvantages of using a data-based approach are high computational costs in the inference mode and challenging interpretability. For these reasons, data-based methods are less useful for classification tasks [20].

### 3.2. Feature-Based Approach

The feature-based approaches assume two stages:

1. A sequence-dependent feature-extraction (FE) stage that transforms time-series into numerical features that can be processed, while preserving the information in the original data set. It yields better results than applying machine learning directly to the raw data.
2. Feature-based classification of the resulting numerical features. This stage can also include the process of reducing the number of features required for effective classification.

Feature-based classification typically has a number of advantages, such as a reduced amount of storage and rapid inference time. Moreover, in the case of small feature-spaces, such methods can have a high degree of result interpretability [19,21]. Feature-based approaches may also be less sensitive to complex patterns in the series than data-based approaches [17].

In recent years, a number of different techniques were proposed for time-series FE. Some recent popular FE algorithms implementations are in libraries Catch22 [22], tsfresh [23], TSFEL [24], and hctsa [25], amongst others. Earlier techniques were based on Fourier and wavelet transforms [18]. Note, most of these FE libraries do not include authors’ recommended classifiers; resulting features are typically combined with random forest or logistic regression classifiers [18].

There are also some recent techniques that combine FE with classification. For example, time-series forest [19], random interval spectrum ensemble [17], diverse representation canonical interval forest classifier (DrCIF) [26] and others.

### 3.3. Hybrid Approaches

Some TSC techniques use only time-domain data representation, while others use frequency-based data representation. Consequently, the first techniques are more sensitive to the appearance of patterns in the data, while the other techniques are more sensitive to the period during which these patterns appear. In general, each approach has its own advantages and disadvantages.

Thus, some authors proposed to combine these techniques into ensembles of classifiers, such as HIVE-COTE (and HIVE-COTE V2) [17,26]. These two ensemble-based techniques combine different classifiers that are sensitive to the shape, the period of the shape repetition, time domain features and spectrum domain features. The results for all classifiers are combined with the hierarchical voting and class-agnostic weights that are proportional to the probability of correct classification for each class. Perhaps the most serious disadvantage of this technique is the high training and inference time, which can take a few days or even weeks for large datasets. HIVE-COTE has bi-quadratic time complexity, and another popular technique—time-series combination of heterogeneous and integrated embedding forest (TS-CHIEF) [27]—has quadratic time complexity [21,28].

### 3.4. Deep-Learning-Based Approaches

Many deep-learning-based (DL-based) architectures have been proposed recently for TSC, such as InceptionTime [28], ResNet-1D, siamese networks [29] and others [30]. Most of the modern TSC architectures belong to the family of 1D convolution neural networks (CNNs) or their modifications [31]. With this approach, there is no need for manual FE and series of any complexity and dimension can be processed. However, the inference and training time is long and the method lacks any interpretability of the results.

One of the most notable DL-inspired methods is RandOm Convolutional KErnel Transform (ROCKET). This method is based on a large number of convolutional kernels with random parameters. It shows almost state-of-the-art performance for the UCR benchmark [20], with training times significantly lower than for HIVE-COTE and InceptionTime [32]. The next modification of this method, MiniROCKET, proposed an improved strategy for kernel parameter selection with an improvement in speed and without loss of accuracy [33].

### 3.5. Classifier Selection Discussion

Finding the optimal classifier selection is still an open problem. Some recent progress has been made and some implementations can be found, for instance, using the UEA & UCR Time-Series Classification Repository [34], and sktime [35] and tsai [36] frameworks. However, even if a particular TSC method shows good results in terms of common benchmarks, see [20], this does not guarantee its performance for any specific problem. All mentioned methods suffer either from high complexity (i.e., high computation time) or from working only for some specific domain of time-series. Therefore, it is commonly required to do some additional adjustments, such as feature selection, method selection, or some domain-related modifications.

## 4. Experimental Design

The devices under test (DUT) that were chosen are 22” computer displays of two similar models: Dell P2217H and Dell E2214H. The collected database includes samples taken from a total of 40 displays, 20 per device model.

### 4.1. Electrical Setup

**Figure 3:**
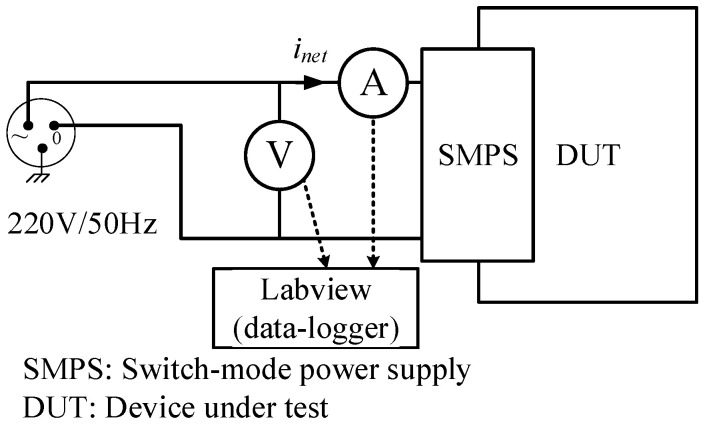
Schematic diagram of the experimental setup.

The electrical measurement setup (Figure 3) is based on a common electrical network with a nominal network voltage of 220–240 $V_{rms}$ and a standard network frequency of about 50 Hz. Each electrical outlet has its own impedance due to branching cables, impedance mismatches and other factors. To diminish the influence of outlet impedance, all the measurements were performed with the same outlet. The consumed current levels were sampled by a digital ammeter (NI-9227 in cRIO-9082 chassis) and data-logged. The sampling was set to 24-bit resolution and a rate of 50 kS/sec. The sampling frequency was set far beyond the electrical network frequency due to the harmonic nature of the current signal [37]. The sampling and data logging was controlled by Labview software (NI DAQExpress). Further analysis was performed by dedicated Python-based software.

### 4.2. Data Collection

Each sampled signal included a 250-s recording at a 50 kHz sampling frequency with a total of $1.25\times 10^{7}$ samples. Two signals for each device were sampled independently and sequentially with a 5-s delay between them to provide independent train and test parts. Manual triggering was applied.

### 4.3. Database

To create the signal database, all the samples were divided into non-overlapping segments with a length of 10,000 samples, which are 20 ms of the signal. Each segment is labeled with a class in accordance with its device number (0 to 39). Thus, the database has 50,000 entries of 10,000 samples (1250 entries per class), both for the train and the test parts.

### 4.4. Experimental Assumptions

In the following, we outline the experimental assumptions of our experiment. First, at least 15 min of “heat up” time was provided in order to provide steady-state conditions for all the devices. All devices had similar settings, e.g., without anomaly differences in brightness and contrast. During the experiment, the nearly same temperature was continually maintained by an air-conditioner.

## 5. Evaluation

### 5.1. Preliminary Analysis

**Figure 4:**
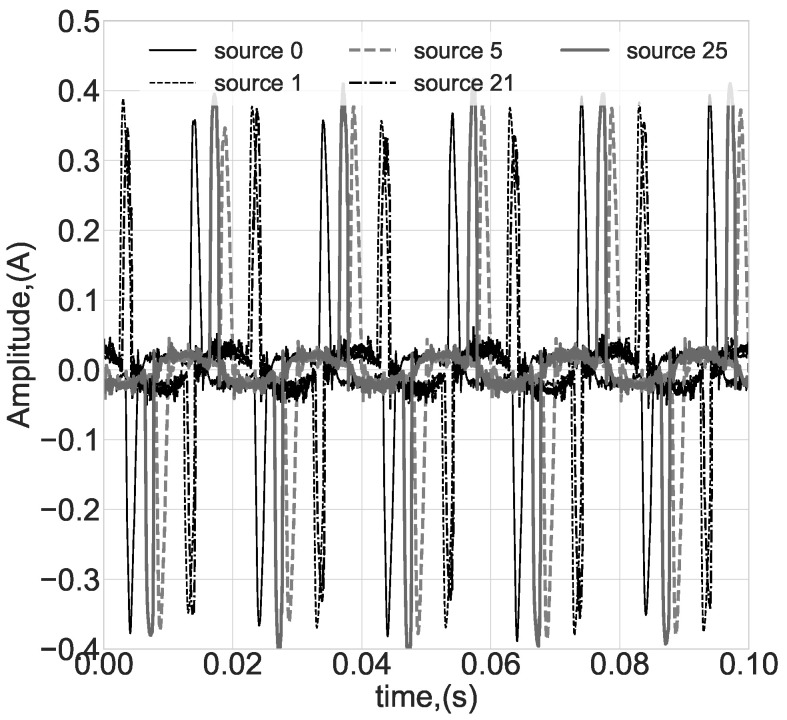
Typical segments of data for some sources (classes).

Preliminary analysis showed that the resulting signals are nearly stationary in time-domain, and without trends and significant changes in frequency components. These results suggest concentrating on time- or frequency-behavior similarity for each class instance, discarding in-shape or pattern similarity classifiers. A sample plot of the segment examples is presented in Figure 4.

### 5.2. Feature-Extraction

In this section, describe the three feature-extraction techniques that were used to derive signal features. In the following section, these features are fed into different classifiers.

#### 5.2.1. TSFEL

TSFEL (Time-Series Feature-Extraction Library) [24] was used as a sample of a fast and accurate FE framework. In the TSFEL framework, all features are extracted in an unsupervised way. The extracted features correspond to the summary statistics in the time- and frequency-domains, including Fourier and wavelet transforms. A set of 390 common features [38] was created from the data. For the wavelet decomposition, the Mexican hat wavelet with 1–10 orders (widths) was applied.

#### 5.2.2. MiniROCKET

The MiniROCKET algorithm [33] was taken on the base of tsai [36] implementation. The underlying transformation includes about 2000 random kernels with different parameters and dilation values. The particular implementation details are provided in Appendix A.1. Since the performance of the MiniROCKET algorithm depends on a set of random parameters, the classification performance has high variability, which leads to weak reproducibility. For this estimator, the best performance among 10 experiments is presented (Section 5.4).

#### 5.2.3. Empirical Wavelet Transform (EWT)

In the EWT method, empirical wavelet filters adopt the use of adaptive partitions of the Fourier domain in order to create an accurate time-frequency representation [39]. EWT is a popular technique for signal FE. The particular implementation details are provided in Appendix A.2.

The additional selected feature-space includes summary statistics (showing the energy characteristics of each time-series in the time domain), an auto-regression coefficient, a barycenter frequency and a correlation-based point-wise frequency (showing frequency-domain summary statistics). We also note that the obtained feature-space is expected to be exhaustive, and some of the features can have a higher correlation, but we assume this effect is corrected during the feature selection stage.

### 5.3. Feature Classification

After using the above-mentioned FE methods, the following standard classifiers were applied:

- Logistic regression (LR) classifier;
- Random forest (RF) classifier with Gini-index-based splitting criteria, ensemble of 100 classifiers and unlimited tree depth;
- LDA classifier with a pre-selected tolerance threshold for singular values of data decomposition (SVD). The threshold was selected using a grid search in the range from $10^{-5}$ up to 1. This search was done because we had noticed the significant influence of the tolerance threshold value on the obtained results;
- Naive Bayes (NB) classifier;
- k-nearest neighbors (kNN) classifier with k = 1 (1-NN). This classifier was used as a baseline due to its relatively high computational time and low classification accuracy.

All implementations of these classifiers were taken out-of-the-box from the scikit-learn Python package.

### 5.4. Evaluation Results

This section presents the evaluation results of different feature extraction methods combined with different classifiers. The resulting classification accuracy for test data for all the evaluated methods is summarized in Table 1. The table presents four feature-selection options with the corresponding number of features for each feature extraction and feature-selection method:

1. Full feature-space of the feature extraction method.
2. Reduced feature-space with feature selection by correlation coefficient. Features with a correlation coefficient of 0.95 or higher were removed (*cor.select*).
3. The previous feature subset further reduced by random-forest feature selection, i.e., selection by feature importance with threshold values 20% of importance (*cor.+rf*).
4. Reduced feature-space only by random-forest feature selection (*rf select*).

**Figure 5:**
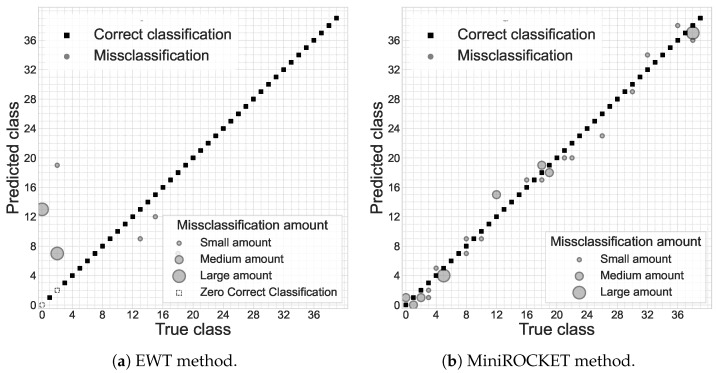
Confusion plots for the best results for EWT-based (**a**) and MiniROCKET-based (**b**) approaches; medium amount ∈[100,600); large amount ∈[600,1250).

Figure 5 presents the confusion matrices for the two most accurate algorithms (marked with bold in Table 1). In all cases, misclassifications were between devices of the same model without misclassifications between models.

## 6. Discussion

### 6.1. MiniROCKET

MiniROCKET method was shown to have the best classification performance while deriving the smallest number of features. Since the method is based on random initialization and highly depends on initialization parameters, only the best accuracy among 10 evaluations is presented. Note, most of the evaluations have mediocre results. Moreover, different MiniROCKET classification evaluations made mistakes on different segments. To conclude, while having the highest ’lucky-shot’ accuracy, these results are hardly repeatable.

### 6.2. TSFEL and Empirical Wavelet Transform

Both the TSFEL- and EWT-based approaches produced similar, but easier to reproduce results when compared to MiniROCKET. While TSFEL has a relatively small feature space, the proposed EWT-based approach provides the most tractable results, since it depends on frequency bands division and summary statistics for each band.

The results in Figure 5 show that the misclassifications for several particular devices are the most significant; most of the devices were classified with 100% accuracy. However, the EWT-based classifier fully misclassified two sources. This problem can probably be solved by some different feature-space expansion or by adding frequency bands.

### 6.3. General Aspects

The analyzed data was archived in a fully unsupervised mode of operation for the same type of device under similar conditions. Consequently, the signals did not differ much. Nevertheless, it was possible to tell which device was which. Furthermore, while the applied algorithms are among the most accurate TSC methods and use a large feature space, their overall computational complexity is expected to be low enough for IoT implementation (e.g., [34,40,41]).

The evaluation included two similar groups of identical devices. All the misclassifications in Figure 5 are among identical devices without misclassifications between devices from different groups. This result can be explained by significant differences even between similar models.

## 7. Conclusions

The main goal of the current study was to determine the possibility of distinguishing a specific device from several same-model devices using only its consumed current. The study may be thought of as a part of a suite of methods for passive device identification, i.e., device fingerprinting under arbitrary conditions. In particular, the main issue related to the subject of research is that all devices have exactly the same hardware, the same software and the same unsupervised mode of operation. The overall analysis of the obtained suggests using either an ensemble of MiniROCKET-based models or the EWT-based approach as proposed in this paper. Among all the evaluated classifiers, LDA seems to be the best choice.

The proposed results could serve as a baseline for further research with additional TSC methods, and additional models of evaluated devices. Moreover, it was not examined whether additional parameters such as device aging, temperature fluctuations, different impedance and others would affect identification performance.

The most interesting future implication is the simultaneous fingerprinting of two or more devices on the same electrical line under variable experimental parameters.

## Figures and Tables

**Table 1:** Accuracy of classification.

Method	Feature-Space	LR	RF	LDA	NB	1-NN
TSFEL	390 features	0.63	0.85	0.91	0.84	0.63
	cor.select (248)	0.59	0.78	0.87	0.83	0.59
	cor.+rf (30)	0.65	0.85	0.89	0.84	0.70
	rf select (24)	0.63	0.93	0.91	0.91	0.83
0]1.4cmMini ROCKET	1924 features	0.67	0.84	0.81	0.72	0.69
	cor.select (435)	0.59	0.76	0.80	0.70	0.67
	cor.+rf (18)	0.65	0.90	**0.94**	0.85	0.78
	rf select (19)	0.63	0.92	0.88	0.92	0.88
0]*EWT	2658 features	0.65	0.88	**0.94**	0.81	0.57
	cor.select (781)	0.57	0.64	0.92	0.57	0.54
	cor.+rf (19)	0.56	0.89	0.89	0.88	0.66
	rf select (145)	0.88	0.92	0.92	0.91	0.75

## Data Availability

The repository of the code corresponding to the paper is available at GitHub through the following link: https://github.com/MVRonkin/Passive-Fingerprinting-of-Same-Model-Electrical-Devices-by-Current-Consumption (accessed on 2 January 2023). The corresponding raw data is available as DataPort database https://dx.doi.org/10.21227/zjn8-gw21 (accessed on 2 January 2023).

## Appendix A. Configuration Details

### Appendix A.1. MiniROCKET Configuration

For the FE, about 1932 kernels were generated with the following parameters, recommended by authors of the original paper [33]:

- kernel size set 9;
- kernel weights are initialized with values −1 and 2 in proportion 2:1, so as to have a sum of values equal to 0 (at all 84 kernels);
- kernel dilation rates from 1 to 903 with algorithmically increasing steps (at all 23 values, but 21 rest unique with float32 precision);
- kernel padding calculated as ⌊(k−1)*d)/2⌋, where *k* is the kernel size; *d* is the dilation rate; ⌊⌋ is the floor (integer part) operation.
- the bias values taken during training are [0.25,0.5,0.75] quantiles from the kernel acting (convolution) output for one randomly selected example. Either just one quantile or more quantiles could be used.

The proportion of positive values (PPV) feature is calculated for the output of the kernel acting.

### Appendix A.2. EWT-Based FE

The particular realization of EWT-based FE consists of two steps. First, sequence decomposition was performed as follows.

1. For all segments, the auto-covariance function was calculated and 19 peaks were selected using a common find-peaks routine with an adjusted peak value threshold and peak-peak distance.
2. Start and stop cutting frequencies for filtration bands were determined as middle points between peak positions. For all these bands, we take bands that include one peak, then two peaks, and so on.
3. Filtration is implemented by a rectangular window in the frequency domain. The same filtering, but mirrored and shifted on one point to the left, is performed for a range from $f_{s}/2$ up to $f_{s}$ in order to avoid Hilbert filtration.

For each band, the extracted feature-space consists of the following:

- band summary statistics (mean value, standard deviation, kurtosis, skewness, median value);
- absolute values of band summary statistics (mean value, standard deviation, kurtosis, skewness, median value);
- first-order autoregression coefficient for band;
- variance of residuals from 1-order autoregression for band;
- barycetner frequency, calculated as
  $$\hat{f}=\frac{\sum_{k=0}^{N/2-1}S\left[k\right]f_{k}}{\sum_{k=0}^{N/2-1}S[k]},$$
  where $\hat{f}$ is the estimated frequency, S[k] is the *k*-th value of the DFT of segment s[n], N/2 corresponds to the highest positive frequency of the transform and $f_{k}=f_{s}k/N$ is the *k*-th value of the frequency of a signal sampled with frequency $f_{s}$;
- correlation-based point-wise frequency, calculated as
  $$\hat{f}=\frac{f_{s}}{2\pi }\arccos\left(\frac{\sum_{n=1}^{N-1}s(n)\cdot s(n-1)}{\sum_{n=0}^{N-1}{\left|s\left(n\right)\right|}^{2}}\right).$$

The implementations of barycetner frequency and correlation-based point-wise frequency are taken from the dsatools Python library [42,43].
